# Discussions of Discovery and Translation with Jennifer Barton

**DOI:** 10.1117/1.BIOS.2.2.020501

**Published:** 2025-04-07

**Authors:** Travis Sawyer

**Affiliations:** a University of Arizona, College of Optical Sciences, Tucson, Arizona, United States

## Abstract

Jennifer Barton discusses her pioneering work in optical imaging, in conversation with Travis Sawyer for Biophotonics Discovery.

**Figure:**
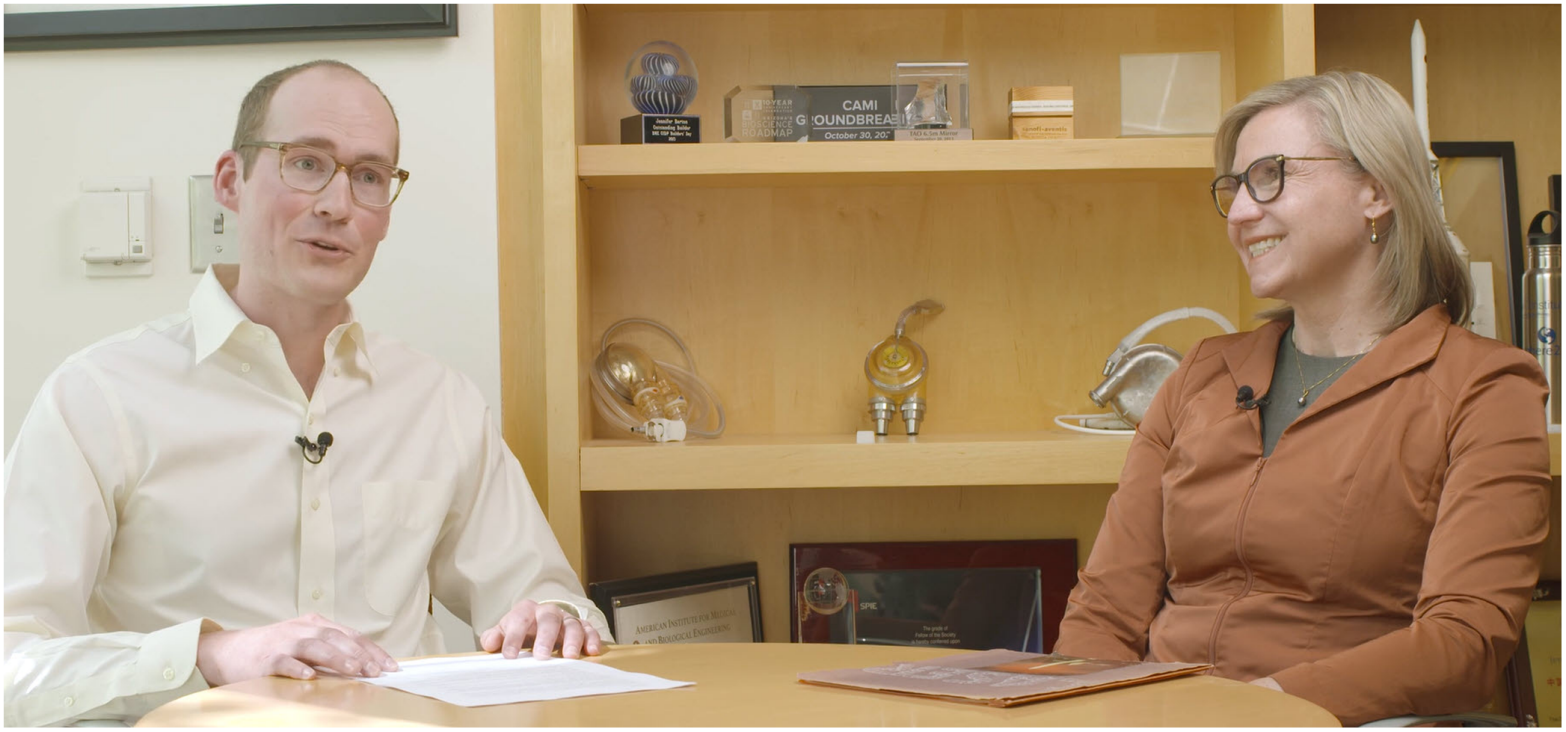
(Left) Travis Sawyer interviewed Jennifer Barton (right) at the University of Arizona, about her pioneering work in biophotonics. View a video recording of the interview at https://doi.org/10.1117/1.BIOS.2.2.020501.

**Figure:**
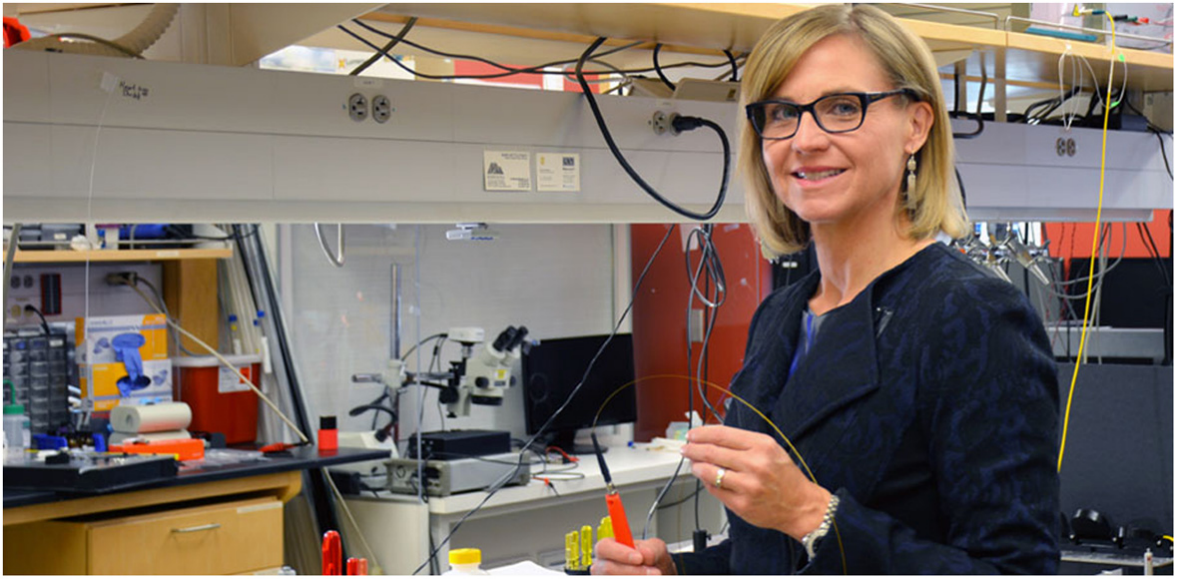
Jennifer Barton is director of the University of Arizona’s BIO5 Institute. BIO5 brings together researchers from across bioscience disciplines – agriculture, bioengineering, biomedicine, pharmacy, and basic science – to develop creative, bold solutions to humanity’s most pressing health and environmental challenges. Credit: Deanna Sanchez.

**Travis Sawyer** is an assistant professor at The University of Arizona College of Optical Sciences and leads the “Biomedical Optics and Optical Measurement” Laboratory. His laboratory’s research interests include biomedical optics, focusing on cancer screening, surgical guidance, and brain imaging.

